# Withdrawal Notice

**DOI:** 10.1002/cam4.5306

**Published:** 2022-11-13

## Abstract

Withdrawal Notice: Zhu, Y, Pu, Q, Zhang, Q, et al. Selenium‐binding protein 1 inhibits malignant progression and induces apoptosis via distinct mechanisms in non‐small‐cell lung cancer. Cancer Med. 2022; 00: 1‐22. doi: 10.1002/cam4.5306. The above article, published online on 13th November 2022 in Wiley Online Library (https://onlinelibrary.wiley.com/doi/10.1002/cam4.5306), has been withdrawn by agreement between the journal Editor in Chief, Dr Stephen Tait, the Authors, and John Wiley & Sons, Ltd. The withdrawal has been agreed due to an editorial office error that led to the publication of the article without peer review. The revised article, which has undergone peer review may be read here: https://onlinelibrary.wiley.com/doi/10.1002/cam4.6309.

